# Development and validation of a nomogram for predicting immune-related thyroid dysfunction during immunotherapy in non-small cell lung cancer: a prospective cohort study in China

**DOI:** 10.3389/fimmu.2025.1611956

**Published:** 2025-07-25

**Authors:** Qianjie Xu, Yuliang Yuan, Xiaosheng Li, Lulu Wang, Wei Zhou, Zuhai Hu, Haike Lei, Yongzhong Wu

**Affiliations:** ^1^ Chongqing Cancer Multi-omics Big Data Application Engineering Research Center, Chongqing University Cancer Hospital, Chongqing, China; ^2^ Chongqing Key Laboratory of Translational Research for Cancer Metastasis and Individualized Treatment, Chongqing University Cancer Hospital, Chongqing, China; ^3^ Department of Health Statistics, School of Public Health, Chongqing Medical University, Chongqing, China

**Keywords:** ICIS, immune-mediated thyroid dysfunction, nomogram, risk model, NSCLC

## Abstract

**Background:**

Immune checkpoint inhibitors (ICIs) have improved survival for non-small cell lung cancer (NSCLC) patients, but immune-related adverse events (irAEs), like immune-mediated thyroid dysfunction (IMTD), have been reported. IMTD causes irreversible thyroid damage, affecting NSCLC patients’ quality of life. This study aims to explore IMTD risk factors and develop a Nomogram to predict IMTD risk at 6, 12, and 24 months.

**Methods:**

Data from 1,917 NSCLC patients from Chongqing University Cancer Hospital treated with ICIs were randomly split into training (70%) and validation (30%) cohorts. After variable selection, a Nomogram with 11 common clinical variables was built from the training cohort. The validation cohort was used to assess the model comprehensively using the Time C-index, Time AUC, Delong test, calibration curves, and decision curve analysis (DCA) to ensure its clinical effectiveness.

**Results:**

IMTD occurred in 343 (17.89%) patients. Among the 11 model factors, Age (OR = 1.02, 95% CI: 1.01 - 1.04), Female (OR = 1.78, 95% CI: 1.31 - 2.42), Mono (OR = 3.52, 95% CI: 1.72 - 7.17), and TCHO (OR = 1.13, 95% CI: 1.03 - 1.24) were significant IMTD risk factors. WBC and FT4 were protective factors (OR = 0.9, 95% CI: 0.83 - 0.98 and OR = 0.94, 95% CI: 0.90 - 0.97). The Nomogram showed good predictive accuracy and generalizability in both cohorts, with C - indices of 0.77 (95% CI: 0.74 - 0.80) and 0.72 (95% CI: 0.67 - 0.78), and AUC values above 0.7. Kaplan - Meier curves confirmed its effective IMTD risk stratification.

**Conclusion:**

The developed Nomogram has good predictive performance and can identify high-risk IMTD patients. The web calculators are user-friendly, providing a basis for early clinical intervention to reduce IMTD incidence.

## Introduction

Non-small cell lung cancer (NSCLC) is experiencing a continuous rise in both incidence and mortality rates, imposing a significant disease burden in China and worldwide (1). Immune checkpoint inhibitors (ICIs) are antibodies that block inhibitory immune regulatory factors, including anti-programmed cell death-1 (PD-1), anti-programmed cell death ligand-1 (PD-L1), and anti-cytotoxic T-lymphocyte antigen-4 (CTLA-4) (2). With the advancement of therapeutic strategies, ICIs have gradually become a standard treatment for NSCLC, offering patients improved survival outcomes (3). Although ICIs effectively modulate immune responses against tumor cells, they may also excessively activate the immune system, leading to immune-related adverse events (irAEs) (4). irAEs can affect nearly all organ systems, but studies have identified immune-mediated thyroid dysfunction (IMTD) as the most common endocrine-related irAEs (5).

IMTD primarily manifests clinically as overt hypothyroidism, overt thyrotoxicosis, subclinical hypothyroidism, and subclinical thyrotoxicosis. The median onset time of IMTD is 6–10 weeks after the initiation of ICI treatment, but cases have been reported as early as 7 days and as late as 3 years (6). The incidence of IMTD varies depending on the type of drug, sex, and ethnicity, ranging from 2.6-50.5% (5, 7, 8). CTLA-4 inhibitors are associated with an IMTD incidence of approximately 2.5% - 5.2%, while PD-1/PD-L1 inhibitors result in an incidence of 3.9% - 8.5%. Combination therapy with PD-1 and CTLA-4 inhibitors leads to a higher incidence, ranging from 10.2% - 16.4% (9, 10). Studies have shown that nearly half of IMTD patients develop irreversible thyroid damage, necessitating lifelong hormone replacement therapy, which poses a significant challenge to improving the quality of life in NSCLC patients (11). However, there is currently a lack of simple and convenient tools to assist clinicians in quickly assessing IMTD risk levels. Existing studies have identified risk factors such as female sex, age, White or Black ethnicity, and prolonged treatment duration. However, these factors are generally non-modifiable, and there is limited research on modifiable risk factors for IMTD (12, 13). Therefore, investigating IMTD risk factors and developing reliable models to accurately predict the risk of IMTD in NSCLC patients undergoing ICI therapy is of great clinical significance.

The nomogram model based on Cox regression is a user-friendly and powerful graphical tool that integrates biological and clinical variables, visualizing the relationships between multiple factors. It is widely used as a clinical prediction model (14).

In summary, this study aims to explore the risk factors for IMTD in NSCLC patients treated with ICIs using Cox regression analysis and develop a visualized nomogram model to predict the risk of IMTD at 6, 12, and 24 months. This model is expected to provide clinicians with a rapid and effective tool for IMTD risk stratification, aiding in formulating personalized treatment strategies and minimizing the adverse effects of IMTD on patients.

## Materials and methods

### Data source

This study comprehensively collected data from 2,123 NSCLC patients who received ICI therapy at Chongqing University Cancer Hospital between January 1, 2019, and December 31, 2023. The collected data included sociodemographic characteristics, such as sex, age, and body mass index (BMI). Tumor-related information, including Karnofsky Performance Status (KPS) and TNM stage. Hematological parameters, including white blood cell count (WBC), hemoglobin (Hb), neutrophils, monocytes (Mono), platelet count (PLT), albumin (ALB), globulin (GLB), triglycerides (TAG), total cholesterol (TCHO), low-density lipoprotein (LDL), high-density lipoprotein (HDL), creatinine (Cr), uric acid (UA), and glucose (GLU). Thyroid-related biomarkers, including serum-free thyroxine (FT4), thyroxine (T4), serum-free triiodothyronine (FT3), triiodothyronine (T3), anti-thyroid peroxidase antibody (TPOAb), thyroid-stimulating hormone (TSH), thyroglobulin (Tg), and thyroglobulin antibody (TGAb). All blood tests were conducted in the Chongqing University Cancer Hospital Laboratory. The follow-up period will continue until January 31, 2025.

### Inclusion and exclusion criteria

**Figure 1:**
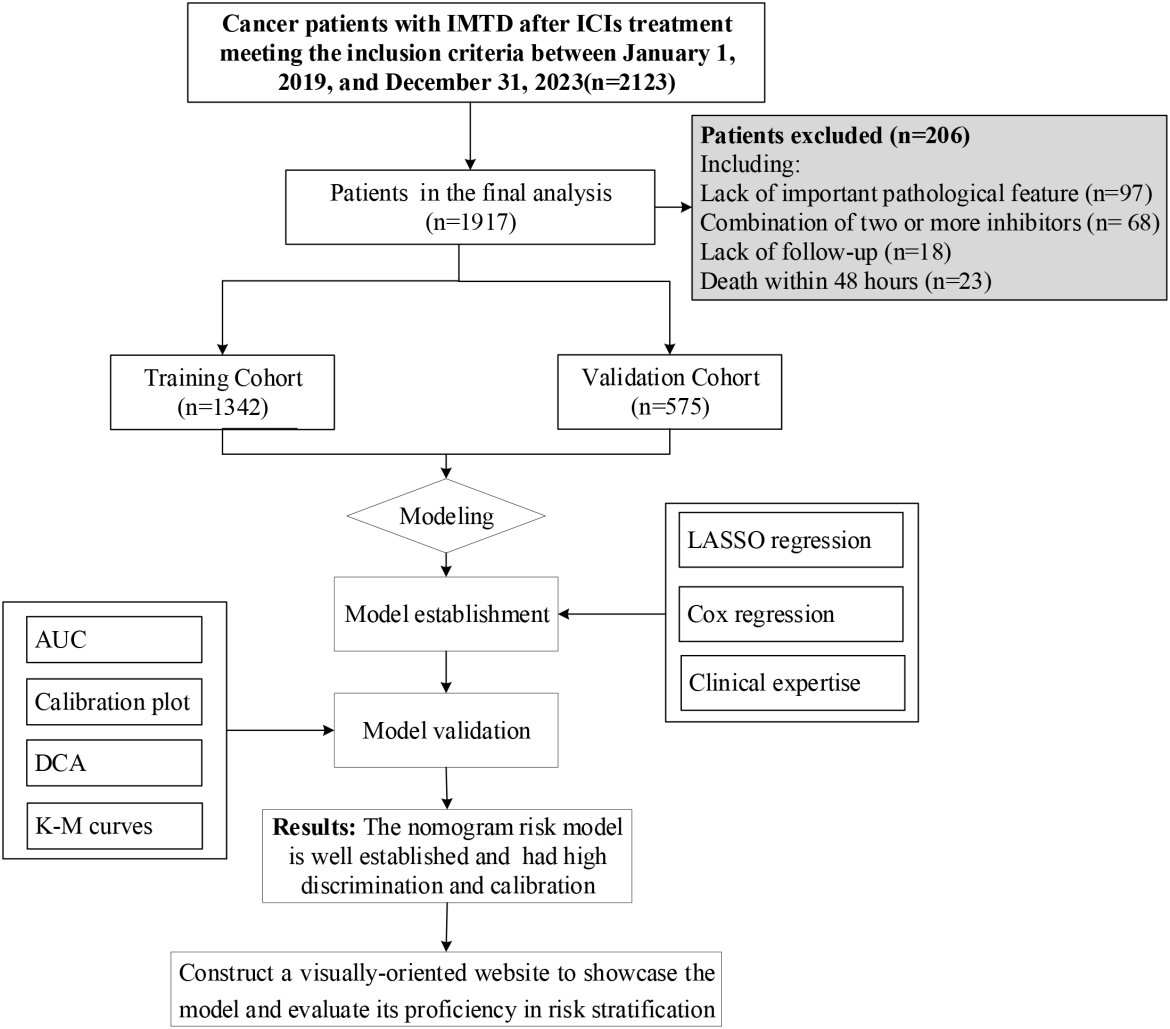
Flow chart of the patients enrolled in the final study cohorts.

The inclusion criteria were as follows: (1) Age ≥18 years; (2) Diagnosis of NSCLC and at least one hospitalization; (3) Received ICI therapy with at least one of the following inhibitors: CTLA-4, PD-1, or PD-L1. The exclusion criteria included: (1) Missing key pathological data, such as thyroid-related biomarkers; (2) Death within 48 hours of hospital admission; (3) Pre-existing thyroid dysfunction before ICI therapy; (4) Combination therapy with two or more ICIs inhibitors; (5) Lack of follow-up data. After applying the inclusion and exclusion criteria, 1,917 patients were included in the model construction, as illustrated in **Figure 1**.

### Definition of IMTD

The diagnosis of immune-mediated thyroid dysfunction (IMTD) in this study was based on previously established criteria (15): i) Overt thyrotoxicosis: TSH < 0.27 mIU/mL and FT4 > 22.00 pmol/L. ii) Subclinical thyrotoxicosis: TSH < 0.27 mIU/mL and 12.00 pmol/L ≤ FT4 ≤ 22.00 pmol/L. iii) Overt hypothyroidism: TSH ≥ 10.0 mIU/L, regardless of FT4 levels. iv) Subclinical hypothyroidism: 4.2 mIU/L < TSH ≤ 10.0 mIU/L and 12.00 pmol/L ≤ FT4 ≤ 22.00 pmol/L.

### Model construction and validation

This study randomly divided patients meeting the inclusion and exclusion criteria into a training cohort (n = 1,342) and a validation cohort (n = 575) in a 7:3 ratio. This randomization was performed using the “caret” package in R with a fixed random seed to ensure result reproducibility. In the training cohort, variable selection was performed through LASSO regression, stepwise multivariate Cox regression, and clinical expertise. The selected variables were then incorporated into a Cox regression analysis to identify independent risk factors for IMTD and construct a Nomogram model. At the same time, we use the “DynNom” package to make the Nomogram model a user-friendly web calculator, allowing anyone to use the website. The performance of the Nomogram model was subsequently validated in the validation cohort. Specifically, The “pROC” package was used to calculate the area under the receiver operating characteristic (ROC) curve (AUC) to assess the generalization performance of the Nomogram model. Time-dependent C-index and time-dependent AUC were used to evaluate the model’s predictive accuracy at different time points. Using Delong test to validate the Nomogram model and compare its performance with other variables included in the model in detail. A calibration curve was generated using 1,000 bootstrap resampling iterations via the “caret” package to validate the predictive accuracy of the Nomogram model in both the training and validation cohorts. To assess the clinical utility of the Nomogram model, we performed decision curve analysis (DCA) using the “rmda” package, visually demonstrating the clinical benefits of the model across different threshold probabilities.

### Statistical analysis

In this study, appropriate statistical methods were applied to describe and compare different types of variables. For normally distributed continuous variables, data were presented as mean ± standard deviation (Mean ± SD), and t-tests were used for group comparisons. For non-normally distributed continuous variables, data were presented as median (M) and interquartile range (IQR), and non-parametric tests were used for group comparisons. Categorical variables were presented as frequencies and percentages, and Chi-square tests were used for group comparisons. The above statistical descriptions and comparisons were conducted using the “tableone” package in R. For handling missing data, we employed multiple imputations using the “mice” package in R, which generates complete datasets by establishing multiple imputation models. This approach effectively minimizes the impact of missing data on study outcomes, thereby enhancing data completeness and analytical reliability (16). This study reported the relevant research results in accordance with the TRIPOD guidelines (17). All statistical analyses were conducted in R version 4.1.2 (http://www.r-project.org). A two-sided *P*-value < 0.05 was considered statistically significant, indicating meaningful differences between groups.

## Result

### Baseline characteristics of the study population

In this study, 1,917 patients were enrolled, among whom 343 cases (17.89%) developed IMTD. The median time of IMTD occurrence in this study was 3.03 months. **Table 1** shows that the mean age of all patients was 61.90 years, with a predominance of male patients (83.20%). However, it is noteworthy that the incidence of IMTD was significantly higher in female patients compared to male patients (31.06% vs. 15.24%, *P* < 0.05). More than half of the NSCLC patients had a BMI range of 18.5-23.9, and approximately two-thirds were classified as TNM stage IV. Statistical analyses revealed that patients who developed IMTD had significantly higher levels of KPS, TAG, TCHO, LDL, Cr, Tg, TPOAb, TSH, and TGAb compared to those who did not develop IMTD (all *P*-values < 0.05). Conversely, WBC, Neutrophil, Mono, PLT, Alb, FT4, T4, FT3, and T3 levels were significantly lower in patients with IMTD (all P-values < 0.05). However, no significant differences were observed between IMTD and non-IMTD patients regarding age, TNM stage, BMI, Hb, Glb, HDL, UA, and GLU levels (all *P*-values > 0.05). No significant differences were observed between the cohorts (**Supplementary Table 1**).

**Table 1:** Sociodemographic and clinical characteristics of subjects.

Variable	Overall (n=1917)	Non-IMTD (n=1574)	IMTD (n=343)	*P*
Age	61.90 ± 8.74	61.79 ± 8.64	62.39 ± 9.18	0.253
KPS	80.62 ± 7.64	80.44 ± 7.78	81.43 ± 6.92	0.030
Sex				<0.001
Male	1595 (83.20)	1352 (84.76)	243 (15.24)	
Female	322 (16.80)	222 (68.94)	100 (31.06)	
TNM				0.817
II-III	639 (33.33)	527 (82.47)	112 (17.53)	
IV	1278 (66.67)	1047 (81.92)	231 (18.08)	
BMI				0.672
18.5-23.9	1128 (58.84)	934 (82.80)	194 (17.20)	
24-27.9	549 (28.64)	443 (80.69)	106 (19.31)	
≥28	90 (4.69)	72 (80.00)	18 (20.00)	
<18.5	150 (7.82)	125 (83.33)	25 (16.67)	
WBC	6.95 ± 3.02	7.12 ± 3.07	6.16 ± 2.64	<0.001
Neutrophil	4.71 ± 2.65	4.85 ± 2.68	4.06 ± 2.41	<0.001
Mono	0.62 ± 0.27	0.63 ± 0.28	0.58 ± 0.26	0.002
Hb	123.84 ± 17.65	124.11 ± 17.58	122.60 ± 17.97	0.152
PLT	245.81 ± 97.12	251.89 ± 97.66	217.88 ± 89.57	<0.001
Alb	38.34 ± 5.02	38.46 ± 4.69	37.76 ± 6.31	0.018
Glb	33.60 ± 6.92	33.65 ± 7.04	33.37 ± 6.34	0.501
TAG	1.70 ± 1.10	1.63 ± 0.99	2.02 ± 1.48	<0.001
TCHO	4.94 ± 1.25	4.87 ± 1.13	5.28 ± 1.67	<0.001
LDL	3.04 ± 0.94	3.02 ± 0.90	3.13 ± 1.07	0.048
HDL	1.35 ± 0.40	1.35 ± 0.40	1.35 ± 0.43	0.885
Cr	66.92 ± 18.31	66.51 ± 17.43	68.80 ± 21.85	0.036
UA	324.01 ± 83.83	323.43 ± 83.37	326.65 ± 85.98	0.520
Tg*	8.50 [4.72, 15.30]	8.20 [4.72, 14.10]	11.30 [4.46, 32.80]	<0.001
FT4	14.67 ± 3.90	15.09 ± 3.02	12.72 ± 6.22	<0.001
TPOAb*	29.00 [28.00, 41.40]	28.00 [28.00, 37.98]	42.30 [28.00, 439.70]	<0.001
T4	109.44 ± 36.52	111.08 ± 29.35	101.89 ± 58.66	<0.001
FT3	4.68 ± 1.22	4.79 ± 0.90	4.17 ± 2.09	<0.001
TSH*	1.70 [1.01, 2.96]	1.64 [1.06, 2.57]	3.89 [0.39, 14.55]	<0.001
TGAb	15.00 [1.30, 16.20]	15.00 [1.30, 15.00]	15.00 [2.40, 93.00]	<0.001
T3	1.81 ± 0.58	1.83 ± 0.50	1.73 ± 0.86	0.002
GLU	5.58 ± 1.77	5.57 ± 1.74	5.64 ± 1.87	0.506

*Expressed as median (M) and interquartile range (IQR).

### Nomogram model construction

**Figure 2:**
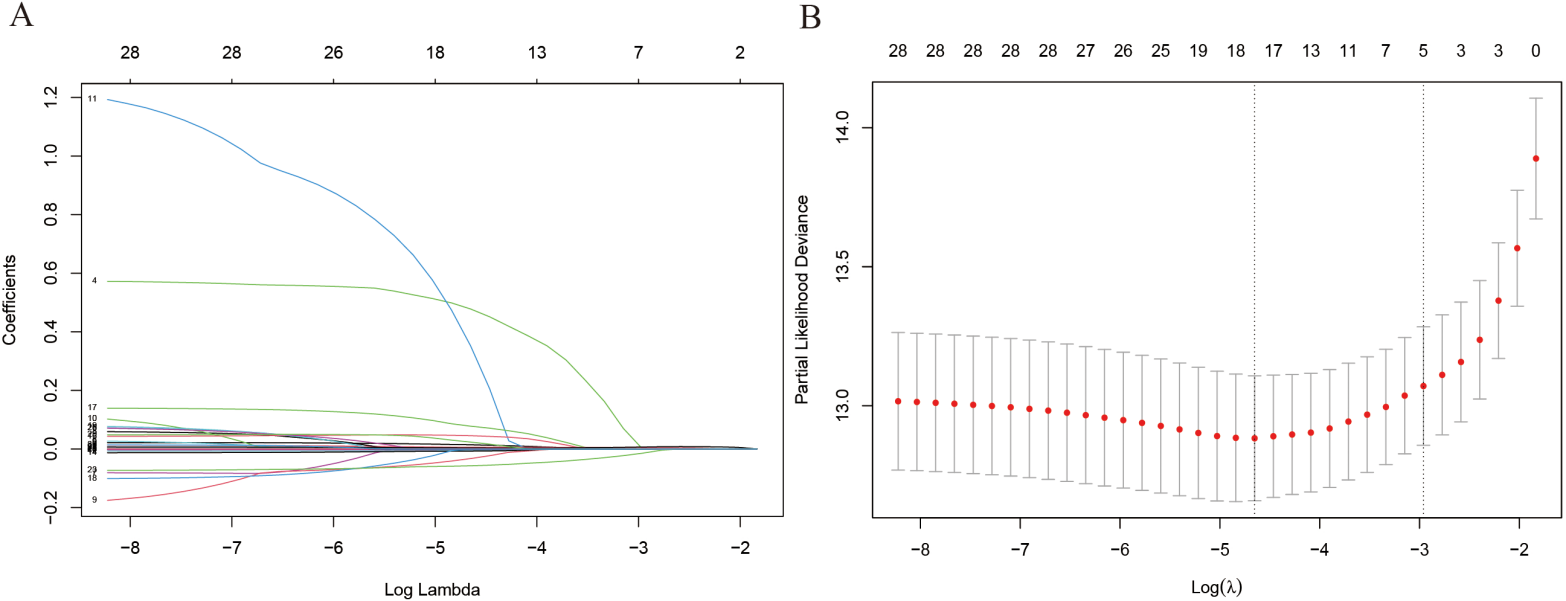
The results of LASSO regress analysis. **(A)** Each curve in the figure represents the changed trajectory of each independent variable coefficient; **(B)** λ value of Lasso regression.

**Figure 3:**
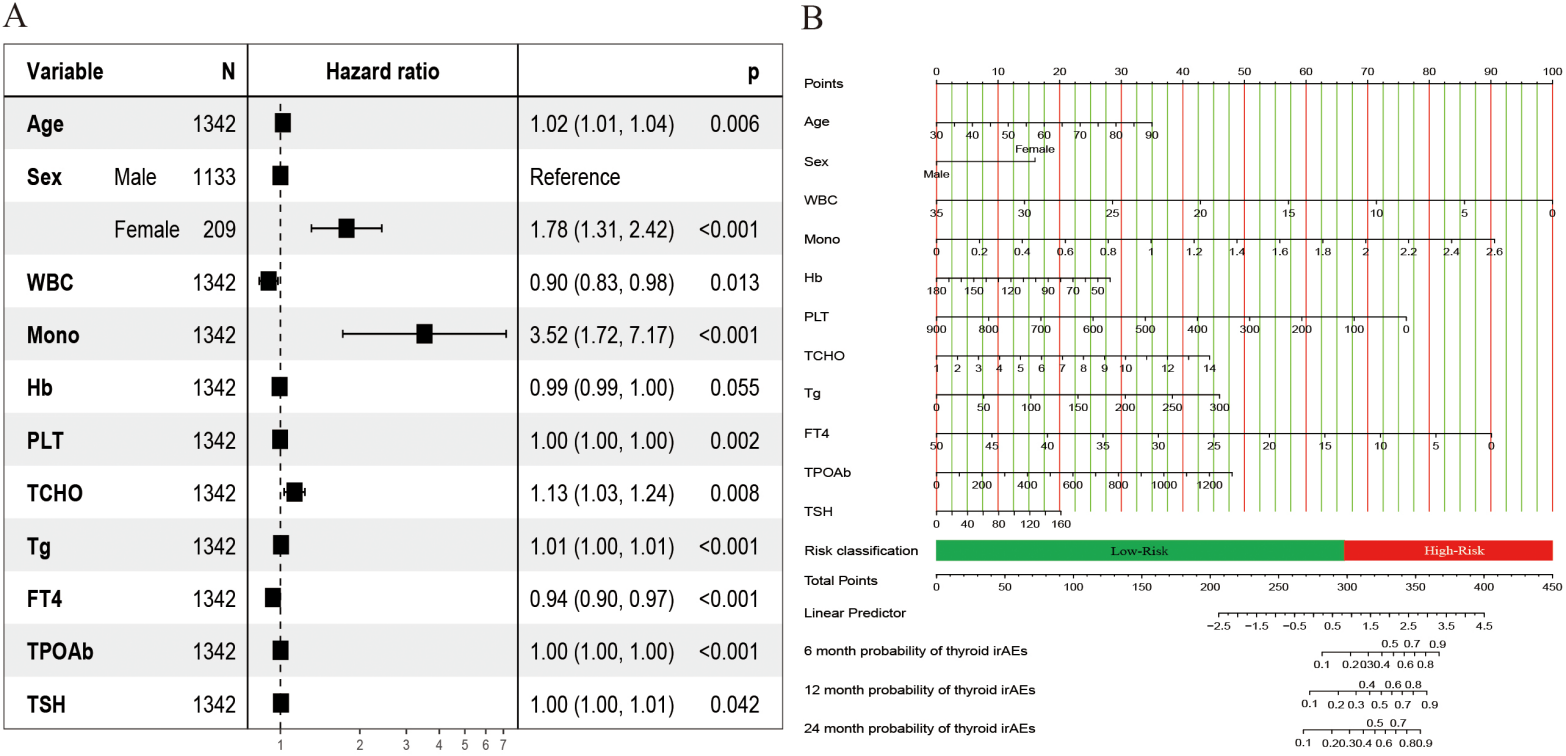
Results of the risk predicted nomogram for IMTD. **(A)** The hazard ratio plot of multivariate Cox regression;**(B)** Nomogram for predicting the 6-, 12-, and 24-month IMTD risk of NSCLC patients after ICI treatment.

**Supplementary Figure 1** shows the correlation of all continuous variables in the study. Following LASSO regression, stepwise multivariate Cox regression, and clinical experience, we identified 11 variables: Age, Sex, WBC, Mono, Hb, PLT, TCHO, Tg, FT4, TPOAb, and TSH to be included in the final model. **Figure 2** shows the results of the LASSO regression analysis, in which we adopted the screening principle of minimizing λ. The hypothesis test results show that the Cox regression model constructed in this study conforms to the proportional hazards hypothesis. **Figure 3A** presents the multivariate Cox regression analysis results for the final variables. The analysis revealed that Age, Female sex, Mono, and TCHO were significant risk factors for IMTD, whereas WBC and FT4 were important protective factors. The remaining variables had minimal impact on IMTD occurrence and were deemed negligible. Mono had the most significant influence among these variables, followed by Sex. Specifically, when all other factors remained constant, each unit increase in Mono level was associated with a 3.52-fold higher relative risk of developing IMTD. Additionally, female patients had a 1.78-fold higher relative risk of IMTD compared to male patients.

**Figure 3B** displays the Nomogram model constructed based on the Cox regression results. A total score is obtained by summing the individual scores assigned to each variable. Based on the nomogram scale, this score is then used to estimate the cumulative probability of IMTD occurrence over different time points. The web-based calculator we developed can quickly calculate the probability of IMTD risk for patients at any time within 24 months. It is accessible via the following URL: https://cqch.shinyapps.io/IMTD/. For example, a 60-year-old male patient has a WBC of 7, Mono of 1, Hb of 116, PLT of 271, TCHO of 5, Tg of 30, FT4 of 16, TPOAb of 156, and TSH of 10. His probability of developing IMTD within 1 year is 0.850 (95% CI: 0.81-0.89).

### Model evaluation and validation

**Figure 4:**
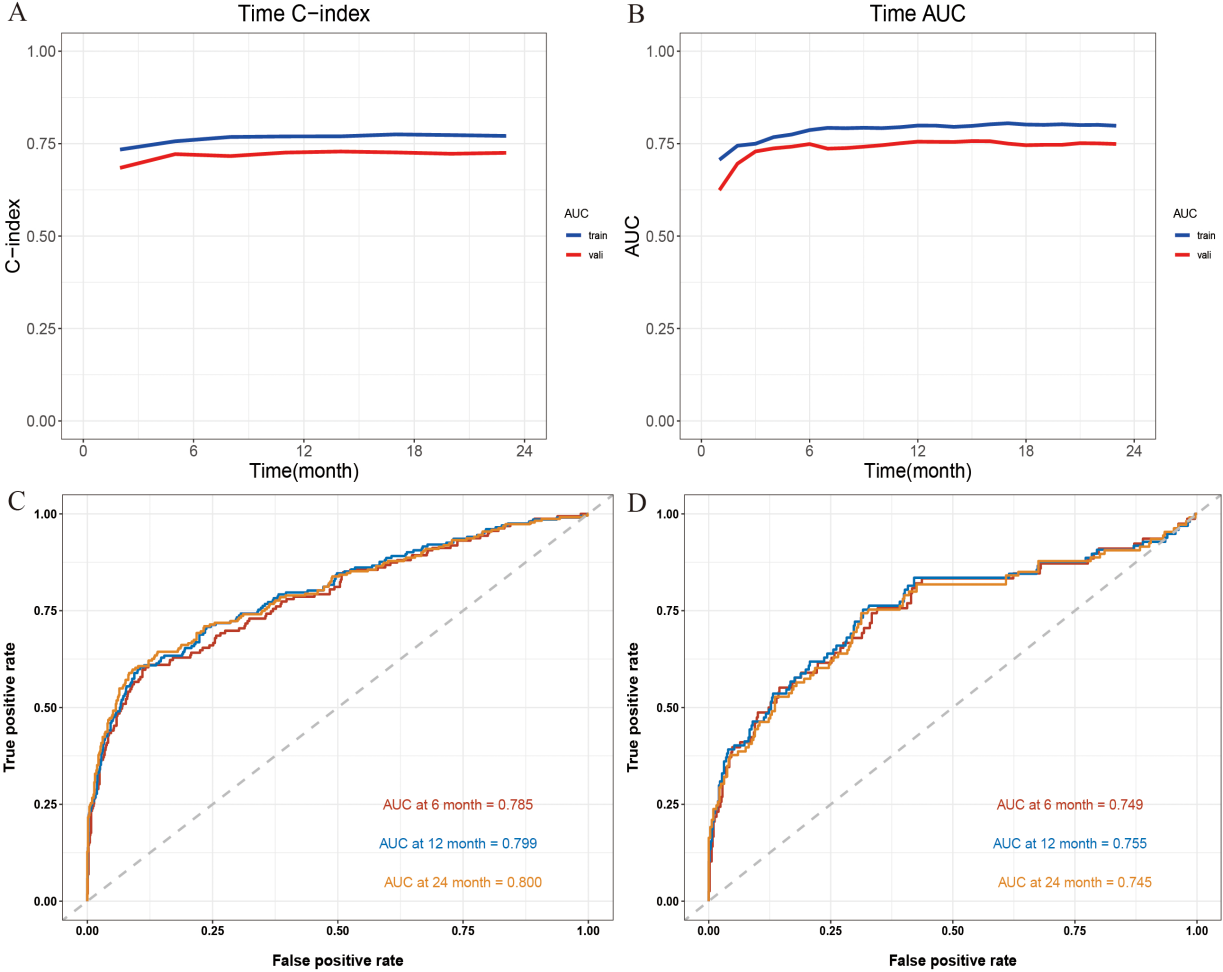
**(A)** The C-index at different time points; **(B)** The AUC at different time points; **(C)** The 6-, 12- and 24-month ROC curves for the Nomogram model in the training cohort; **(D)** The 6-, 12- and 24-month ROC curves for Nomogram model in the validation cohort.

**Figure 5:**
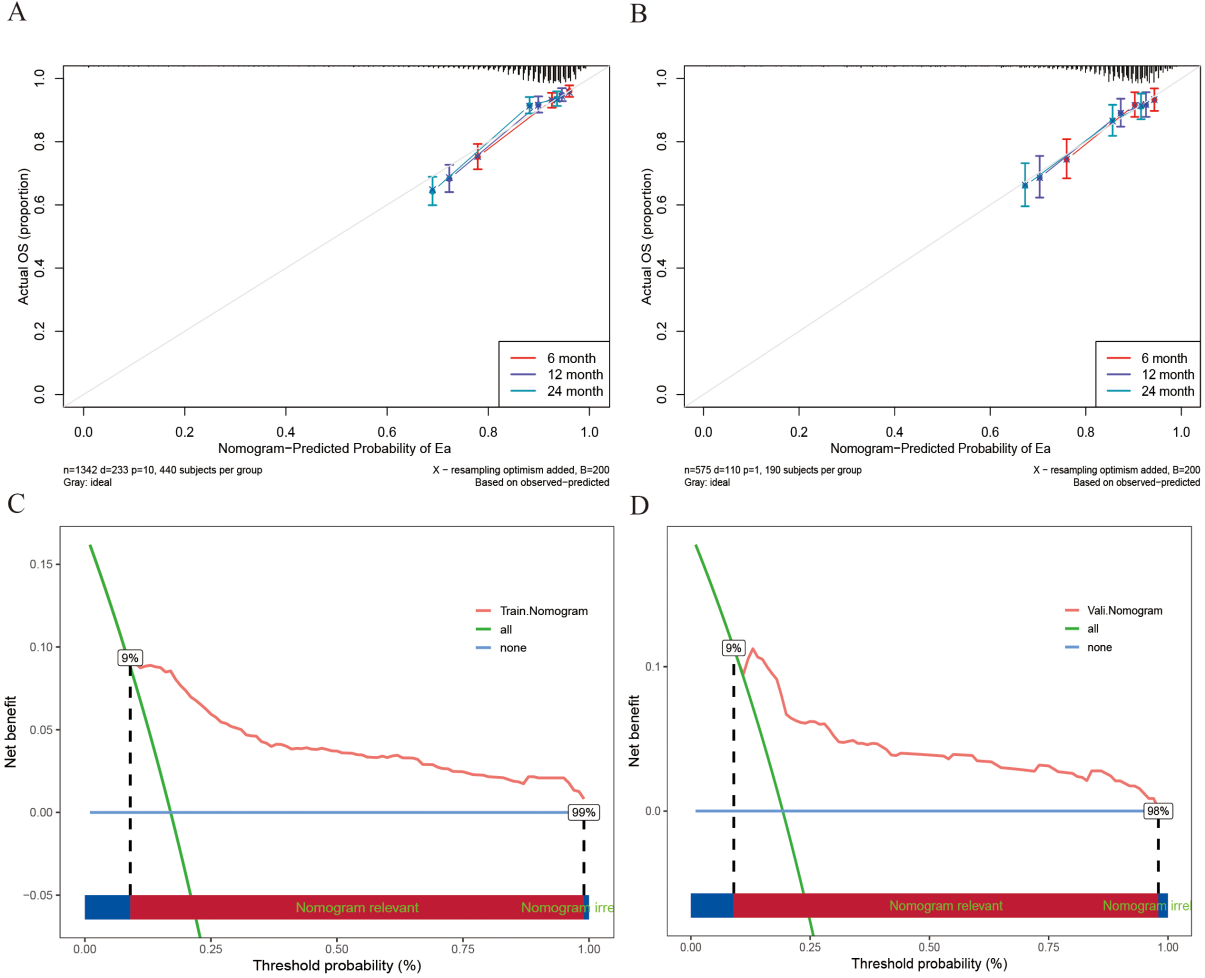
**(A)** The 6-, 12- and 24-month calibration curves of the Nomogram model in the training cohort; **(B)** The 6-, 12- and 24-month calibration curves of the Nomogram model in the validation cohort; **(C)** The DCA curves for Nomogram models in training cohort; **(D)** The DCA curves for Nomogram models in the validation cohort.

To systematically assess the performance of the Nomogram model, we conducted evaluations using a validation cohort. The C-index for the model in the training and validation cohorts was 0.77 (95% CI: 0.74 - 0.80) and 0.72 (95% CI: 0.67 - 0.78), respectively, indicating high predictive consistency. Additionally, **Figure 4A** illustrates the C-index of the Nomogram model at different time points for both the training and validation cohorts, demonstrating that the model maintains accurate and robust predictive performance over time. Similarly, **Figure 4B** presents the area under the curve (AUC) values at different time points, confirming the model’s stability and reliability. In the training cohort, the AUC values for predicting 6-, 12-, and 24-month IMTD occurrence were 0.785, 0.799, and 0.800, respectively, while in the validation cohort, the corresponding AUC values were 0.749, 0.755, and 0.745, suggesting good generalization ability. **Figures 4C, D** provide detailed ROC curve analyses for further validation. **Supplementary Table 2** presents the detailed results of the DeLong test. The results indicate that the differences in AUC values between the nomogram model and the univariate models constructed with individual variables are statistically significant (all *P*-values < 0.05). This suggests that the nomogram, which integrates multiple variables, demonstrates superior predictive performance and is significantly better than models based on a single variable. Regarding prediction accuracy, **Figures 5A, B** display the calibration curves for the training and validation cohorts. The calibration plots show that all points are evenly distributed along the diagonal, indicating a strong agreement between predicted and actual IMTD outcomes and demonstrating high predictive accuracy.

**Figure 6:**
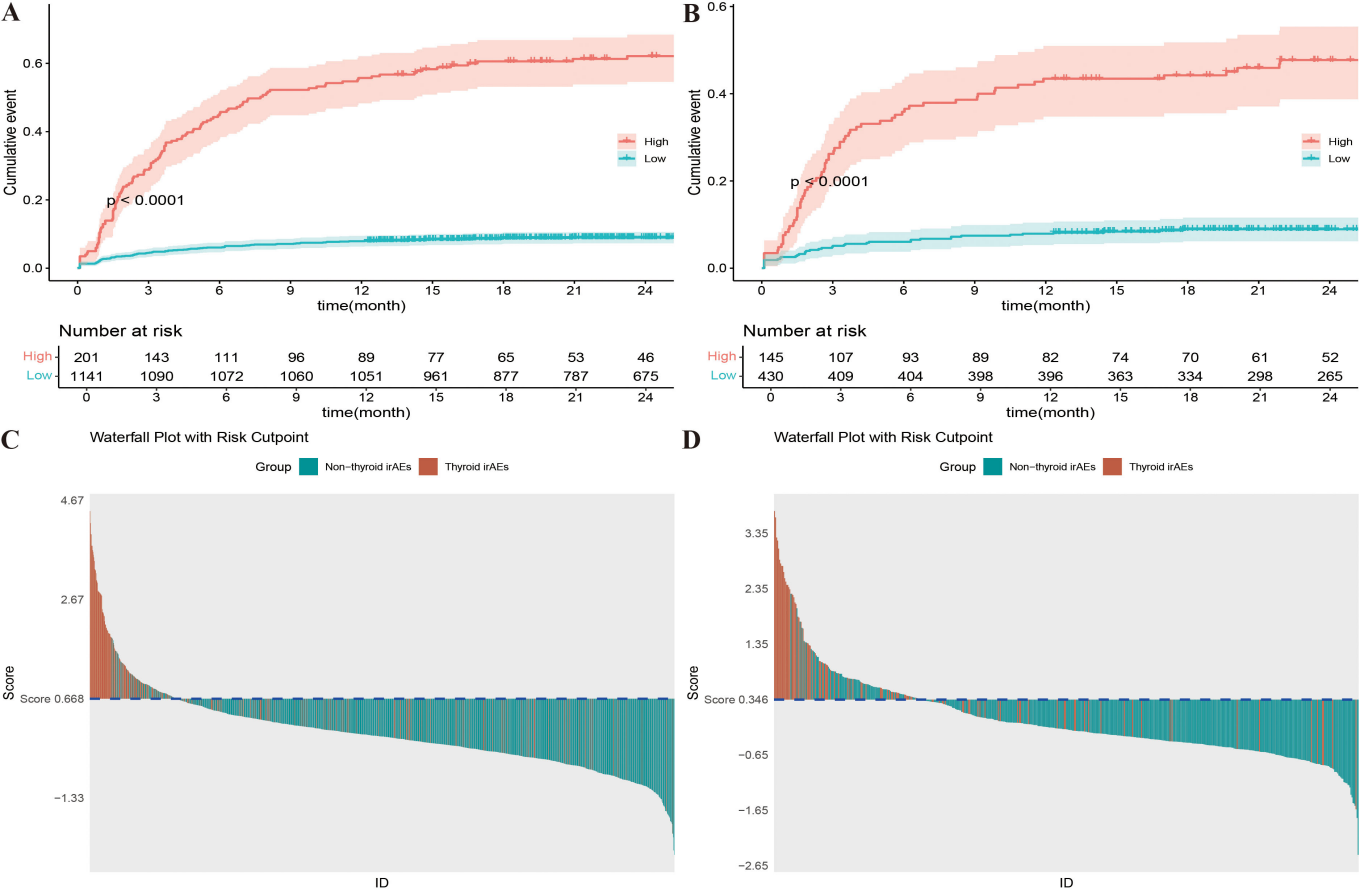
**(A)** K-M curves of different risk levels for Nomogram model in training cohort; **(B)** K-M curves of different risk levels for Nomogram model in validation cohort; **(C)** Prediction results waterfall plot of Nomogram model in training cohort; **(D)** Prediction results waterfall plot of Nomogram model in validation cohort.

Decision curve analysis (DCA) was employed for clinical utility, as shown in **Figures 5C, D**. The results indicate that the model provides a more significant net benefit in the training cohort than the “all” and “none” strategies within a 9% - 99% threshold probability range, confirming its clinical applicability. The model retains clinical value within a threshold probability range of 9-98% in the validation cohort. Regarding risk stratification, **Figures 6A, B** illustrate the risk identification capabilities of the Nomogram model in both cohorts. The model effectively distinguishes IMTD risk levels across different patient groups. Furthermore, **Figures 6C, D** utilize waterfall plots to visualize actual patient outcomes and predicted risk scores across both cohorts, achieving high predictive accuracy in both settings.

## Discussion

ICI therapy has transformed NSCLC treatment and significantly delayed patient mortality. However, with the increased use of ICIs, irAEs have become more common (18). irAEs, caused by an overactive immune response, often affect the endocrine system, with IMTD being a frequently observed type (19). In NSCLC treatment, exploring IMTD risk factors and developing a precise prediction model is crucial for guiding clinical decisions, optimizing ICI therapy, and improving patient outcomes. This study innovatively integrates multi-dimensional data from NSCLC patients treated with ICIs, including demographic characteristics, hematological indicators, and thyroid biomarkers. Through multivariate Cox regression analysis, it not only identifies risk factors associated with IMTD but also develops a new Nomogram prediction model. This model can accurately predict the risk of IMTD at 6-, 12-, and 24 months without additional complex imaging examinations, making it easy to operate and intuitive to understand. It enables clinicians to quickly identify high-risk patient groups and develop personalized care plans, further enhancing the safety of ICI therapy in clinical practice.

Nomograms transform complex Cox regression models into visual graphics, enhancing the readability of prediction results, and are increasingly applied in clinical practice. Kattan et al. compared six machine learning prediction models with Nomogram models based on Cox proportional hazards regression using three large urological datasets and found that Nomogram models had comparable or even superior predictive ability to other machine learning models (20). Similarly, Wang et al. developed a Nomogram model using LASSO-Cox regression to predict recurrence in early-stage hepatocellular carcinoma patients, which performed well (21). In this study, the developed Nomogram model has a C-index and AUC greater than 0.7 within the prediction period, demonstrating excellent predictive accuracy and model robustness.

Several existing models have significantly contributed to predicting thyroid dysfunction during immunotherapy. For instance, Wang et al. developed a thyroid dysfunction prediction model based on hospital electronic medical record system data (8). Our study, however, distinguishes itself by incorporating a wider range of variables, including patients’ sociodemographic characteristics, hematological parameters, and thyroid - related biomarkers. This diversity in variables enables our model to conduct a more nuanced and comprehensive assessment of IMTD risks. Unlike the model by Wang et al., our Nomogram model also offers time - dependent predictions, allowing clinicians to measure IMTD risks at 6, 12, and 24 - month time points. This dynamic approach not only enhances the accuracy of predictions but also equips healthcare providers with crucial information for designing personalized patient management strategies.

To build the final Nomogram model, the study selected 11 variables, including sociodemographic characteristics, hematological indicators, and thyroid biomarkers. Females and Mono were the two most influential factors in IMTD. Existing research has indicated potential gender differences in irAE incidence. Triggianese et al. found that men have a slightly lower incidence of ICI-related endocrine diseases (especially IMTD) than women (22). Niafar et al. noted that women are 5–10 times more likely to develop thyroid-related diseases than men (23). Recent studies have shown that thyroid dysfunction significantly affects blood parameters, and complete blood counts are crucial for diagnosis (24). Specifically, one study highlighted that patients with thyroid dysfunction have significantly higher Mono levels than the normal population (25). This could be because T3 and T4 in thyroid hormones affect blood formation and cell differentiation and cause changes in blood parameters (26).

Similar findings have been reported for other hematological indicators. Cao et al. discovered that IMTD patients have significantly higher PLT levels than healthy individuals (27). In IMTD patients, the abnormal immune system attacks thyroid tissue, affecting platelet production and metabolism through inflammatory factors and anti-thyroid antibodies. Elevated levels of inflammatory factors such as interleukin-6 (IL-6) and Tumor necrosis factor-α (TNF-α) stimulate the proliferation and differentiation of megakaryocytes in the bone marrow, increasing PLT production and leading to higher PLT levels. Anti-thyroid antibodies may cross-react with antigens on PLT surfaces, affecting their metabolism and function and causing PLT abnormalities (28). This situation may further lead to anemia or decreased Hb levels in patients. Research has shown that Hb decline is associated with a higher risk of thyroid dysfunction (29, 30). A cross-sectional study found that blood lipids significantly impact thyroid dysfunction, especially in IMTD. It pointed out that higher TCHO levels increase the risk of thyroid dysfunction (31). This is because NSCLC patients with high TCHO levels often have high leptin levels, which increase TSH secretion and induce IMTD (32). Notably, in this study, WBC was found to be a protective factor that was not previously reported. The mechanisms underlying WBC reduction associated with IMTD involve complex immune regulatory pathways influenced by cancer therapies and tumor microenvironment dynamics. The tumor microenvironment (TME) plays a pivotal role in modulating immune responses. For example, regulatory T cells (Tregs) can suppress effective anti-tumor immunity through various mechanisms, such as cytokine secretion and cell-cell interactions (33). The presence of tumor-associated immune cells, such as macrophages and Tregs, can inhibit immune clearance and alter systemic immune cell populations, which may contribute to the WBC reductions observed in hypothyroid states linked to tumor immunity. Additionally, the immune suppression mediated by B regulatory cells (Bregs) and other immunosuppressive cells can further complicate the immune landscape, potentially leading to decreased WBC counts as part of broader immune dysregulation (34). Furthermore, immune mechanisms involving PD-1/PD-L1 pathways can inhibit phagocytosis and immune clearance, thereby impacting WBC dynamics (35).

Regarding thyroid biomarkers, FT4 and TSH are crucial for diagnosing thyroid dysfunction, as they are the most sensitive biomarkers for assessing thyroid function. The actual reason for the decline in FT4 levels in cases of thyroid dysfunction is not fully understood. However, it may be due to dysregulation of the hypothalamic-pituitary-thyroid axis or the interaction effects of age and gender (36). Studies have shown that FT4 levels change with age and gender, with FT4 levels being slightly lower in individuals aged ≥60 compared to younger and middle-aged populations (37). In this study, the average age of the NSCLC patients was over 60 years, which could explain why FT4 was identified as a protective factor for IMTD in this research. Patients with IMTD have an immune system disturbance, and the TPOAb produced in the body causes damage and death of thyroid cells, reducing the synthesis and release of thyroid hormones. Furthermore, this also leads to increased TSH secretion by the anterior pituitary gland to stimulate the thyroid gland and produce more hormones to maintain normal physiological function (38). TSH plays a significant role both in diagnosing and predicting IMTD. Brilli et al. pointed out that pre-treatment serum TSH levels can help identify high-risk IMTD patients (39). Additionally, Kobayashi et al. used a multivariate logistic regression model to explore risk factors for IMTD after PD-L1 antibody treatment, and their study highlighted that elevated baseline TSH levels are a significant risk factor for IMTD (40). Due to the interaction between TPOAb and TSH, TPOAb is an important predictor of IMTD risk. A cross-sectional study found that TPOAb-positive individuals are at higher risk for potential thyroid damage (41). Similarly, other studies have used TPOAb as a prognostic indicator for thyroid dysfunction, particularly in thyroid diseases caused by immune responses (42). Tg is a dimeric protein produced only by mature thyroid tissue and stored in the follicular lumen. The concentration of Tg in the blood varies among individuals. It depends on factors such as thyroid mass, TSH levels, and the stimulation of the gland by antibodies such as TPOAb, as well as tissue damage (43). Tg is commonly used as a biomarker for thyroid dysfunction during pregnancy and in Graves’ disease; however, in the study by Kurimoto et al., serum Tg levels were also found to help distinguish patients more likely to develop IMTD (44, 45).

The Nomogram model offers a predicted score of IMTD for each patient. Those with a score > 293.99 are classified as high-risk, others as low-risk. We recommend high-risk patients undergo daily thyroid function monitoring in - hospital and every three months post-discharge. They should also consider adjusting the ICIs treatment plan or preparing for early hormone replacement therapy.

This study has several strengths. First, we applied strict inclusion and exclusion criteria, ensuring that patients unsuitable for the study were excluded, thereby guaranteeing the validity of the data. Second, this is the first large-scale study in Western China to investigate the risk factors and predictive model for IMTD in lung cancer patients following ICI treatment, providing valuable insights for future related research.

However, this study also has some limitations. First, as a retrospective study, it has inherent limitations, such as recall bias and recording bias. To address these issues, we plan to design a prospective cohort study to overcome these inherent flaws and validate the clinical applicability of the model. Second, we did not consider the results of patients’ ultrasound examinations and individual genetic data when constructing the model. In future research, we intend to incorporate genetic and imaging data to enhance the assessment of IMTD risk. Although this may raise upfront costs, it is expected to considerably boost the model’s predictive power and offer a more comprehensive risk evaluation. Finally, this is a single-center study, with all ICI patient data derived from the same hospital, which means the predictive model we developed lacks external validation. The model’s generalizability and robustness need further verification. The study’s conclusions apply primarily to patient groups similar to the study cohort. In subsequent studies, we can expand the sample source and use data from other centers to validate the model’s performance.

## Conclusion

This study developed a Nomogram model using 11 features that are easily accessible in clinical practice to predict the risk of IMTD occurrence in NSCLC patients receiving ICI treatment. The model demonstrates high prediction accuracy and strong generalizability with intuitive and straightforward characteristics. It can quickly assist clinicians and patients in identifying the risk of IMTD in clinical practice, making early adoption of preventive and therapeutic strategies possible and ultimately reducing the likelihood of IMTD occurrence.

## Data Availability

The raw data supporting the conclusions of this article will be made available by the authors, without undue reservation.
