# Supplementary material for: Development and validation of a nomogram for predicting immune-related thyroid dysfunction during immunotherapy in non-small cell lung cancer: a prospective cohort study in China
Source: Front Immunol. 2025 Jul 25;16:1611956. doi: 10.3389/fimmu.2025.1611956 (PMC12331586; doi:10.3389/fimmu.2025.1611956)
Supplement: Supplementary file 1 [file Table1.docx]

Supplementary Table1. Baseline characteristics of subjects.

| Variable | Overall(n=1917) | Training cohort(n=1342) | Validation cohort(n=575) | *P* |
| --- | --- | --- | --- | --- |
| Age | 61.90±8.74 | 62.07±8.66 | 61.50±8.91 | 0.188 |
| KPS | 80.62±7.64 | 80.52±7.70 | 80.85±7.51 | 0.393 |
| Sex |  |  |  | 0.134 |
| Male | 1595 (83.20) | 1133 (84.43) | 462 (80.35) |  |
| Female | 322 (16.80) | 209 (15.57) | 113 (19.65) |  |
| TNM |  |  |  | 1.000 |
| II-III | 639 (33.33) | 447 (33.31) | 192 (33.39) |  |
| IV | 1278 (66.67) | 895 (66.69) | 383 (66.61) |  |
| BMI |  |  |  | 0.694 |
| 18.5-23.9 | 1128 (58.84) | 800 (59.61) | 328 (57.04) |  |
| 24-27.9 | 549 (28.64) | 375 (27.94) | 174 (30.26) |  |
| ≥28 | 90 (4.69) | 61 (4.55) | 29 (5.04) |  |
| <18.5 | 150 (7.82) | 106 (7.90) | 44 (7.65) |  |
| WBC | 6.95±3.02 | 6.90±2.88 | 7.05±3.33 | 0.303 |
| Neutrophil | 4.71±2.65 | 4.65±2.51 | 4.84±2.94 | 0.144 |
| Mono | 0.62±0.27 | 0.62±0.26 | 0.63±0.30 | 0.938 |
| Hb | 123.84±17.65 | 123.79±17.84 | 123.97±17.22 | 0.839 |
| PLT | 245.81±97.12 | 242.89±93.35 | 252.61±105.16 | 0.045 |
| Alb | 38.34±5.02 | 38.37±4.94 | 38.27±5.20 | 0.706 |
| Glb | 33.60±6.92 | 33.56±6.87 | 33.68±7.03 | 0.740 |
| TAG | 1.70±1.10 | 1.70±1.14 | 1.70±1.00 | 0.944 |
| TCHO | 4.94±1.25 | 4.97±1.26 | 4.89±1.25 | 0.196 |
| LDL | 3.04±0.94 | 3.03±0.93 | 3.06±0.94 | 0.627 |
| HDL | 1.35±0.40 | 1.36±0.41 | 1.32±0.39 | 0.072 |
| Cr | 66.92±18.31 | 67.24±18.27 | 66.17±18.41 | 0.238 |
| UA | 324.01±83.83 | 326.39±82.20 | 318.44±87.32 | 0.057 |
| Tg* | 8.50 [4.72, 15.30] | 8.44 [4.83, 15.40] | 8.61 [4.53, 15.05] | 0.699 |
| FT4 | 14.67±3.90 | 14.62±3.87 | 14.78±3.97 | 0.408 |
| TPOAb* | 29.00 [28.00, 41.40] | 29.50 [28.00, 41.40] | 28.00 [28.00, 41.00] | 0.332 |
| T4 | 109.44±36.52 | 108.96±35.33 | 110.54±39.17 | 0.386 |
| FT3 | 4.68±1.22 | 4.71±1.21 | 4.62±1.26 | 0.122 |
| TSH* | 1.70 [1.01, 2.96] | 1.77 [1.01, 2.98] | 1.63 [1.02, 2.86] | 0.232 |
| TGAb | 15.00 [1.30, 16.20] | 15.00 [1.30, 15.90] | 15.00 [1.30, 17.10] | 0.865 |
| T3 | 1.81±0.58 | 1.82±0.58 | 1.79±0.59 | 0.366 |
| GLU | 5.58±1.77 | 5.59±1.77 | 5.56±1.75 | 0.793 |

Note：*Expressed as median (M) and interquartile range (IQR).

Supplementary Table2. Delong test of all models.

| Cohort | Model | 1. month   AUC (95%CI) | *P* | 1. Month   AUC (95%CI) | *P* | 1. month   AUC (95%CI) | *P* |
| --- | --- | --- | --- | --- | --- | --- | --- |
| Training cohort | Nomogram | 0.785(0.742-0.828) |  | 0.799(0.761-0.836) |  | 0.800(0.762-0.837) |  |
|  | Age | 0.533(0.485-0.582) | <0.001 | 0.544(0.500-0.588) | <0.001 | 0.535(0.492-0.579) | <0.001 |
|  | Sex | 0.554(0.519-0.590) | <0.001 | 0.569(0.536-0.601) | <0.001 | 0.568(0.536-0.600) | <0.001 |
|  | WBC | 0.590(0.543-0.637) | <0.001 | 0.613(0.570-0.655) | <0.001 | 0.604(0.561-0.646) | <0.001 |
|  | Mono | 0.497(0.448-0.547) | <0.001 | 0.535(0.490-0.580) | <0.001 | 0.546(0.501-0.591) | <0.001 |
|  | Hb | 0.569(0.524-0.614) | <0.001 | 0.559(0.518-0.601) | <0.001 | 0.537(0.494-0.580) | <0.001 |
|  | PLT | 0.611(0.565-0.657) | <0.001 | 0.619(0.578-0.660) | <0.001 | 0.603(0.562-0.645) | <0.001 |
|  | TCHO | 0.588(0.539-0.637) | <0.001 | 0.578(0.534-0.623) | <0.001 | 0.579(0.535-0.623) | <0.001 |
|  | Tg | 0.573(0.516-0.630) | <0.001 | 0.592(0.542-0.643) | <0.001 | 0.609(0.560-0.659) | <0.001 |
|  | FT4 | 0.595(0.534-0.657) | <0.001 | 0.618(0.564-0.673) | <0.001 | 0.637(0.585-0.689) | <0.001 |
|  | TPOAb | 0.689(0.638-0.740) | <0.001 | 0.678(0.633-0.723) | <0.001 | 0.664(0.619-0.709) | <0.001 |
|  | TSH | 0.496(0.429-0.564) | <0.001 | 0.568(0.508-0.628) | <0.001 | 0.599(0.543-0.656) | <0.001 |
| Validation cohort | Nomogram | 0.749(0.681-0.817) |  | 0.755(0.694-0.817) |  | 0.745(0.685-0.806) |  |
|  | Age | 0.495(0.423-0.567) | <0.001 | 0.484(0.419-0.550) | <0.001 | 0.497(0.432-0.563) | <0.001 |
|  | Sex | 0.587(0.531-0.642) | <0.001 | 0.580(0.531-0.630) | <0.001 | 0.597(0.548-0.647) | <0.001 |
|  | WBC | 0.598(0.529-0.668) | <0.001 | 0.598(0.536-0.660) | <0.001 | 0.586(0.524-0.649) | <0.001 |
|  | Mono | 0.550(0.481-0.620) | <0.001 | 0.546(0.483-0.609) | <0.001 | 0.568(0.503-0.633) | <0.001 |
|  | Hb | 0.523(0.453-0.594) | <0.001 | 0.516(0.452-0.580) | <0.001 | 0.489(0.425-0.553) | <0.001 |
|  | PLT | 0.604(0.538-0.670) | <0.001 | 0.607(0.547-0.666) | <0.001 | 0.578(0.518-0.638) | <0.001 |
|  | TCHO | 0.543(0.470-0.616) | <0.001 | 0.527(0.460-0.595) | <0.001 | 0.536(0.470-0.603) | <0.001 |
|  | Tg | 0.530(0.444-0.615) | <0.001 | 0.539(0.462-0.616) | <0.001 | 0.546(0.472-0.620) | <0.001 |
|  | FT4 | 0.547(0.459-0.636) | <0.001 | 0.590(0.510-0.669) | <0.001 | 0.599(0.522-0.676) | <0.001 |
|  | TPOAb | 0.686(0.613-0.759) | 0.108 | 0.663(0.597-0.729) | 0.013 | 0.659(0.595-0.723) | 0.025 |
|  | TSH | 0.461(0.362-0.560) | <0.001 | 0.527(0.438-0.617) | <0.001 | 0.549(0.463-0.634) | <0.001 |


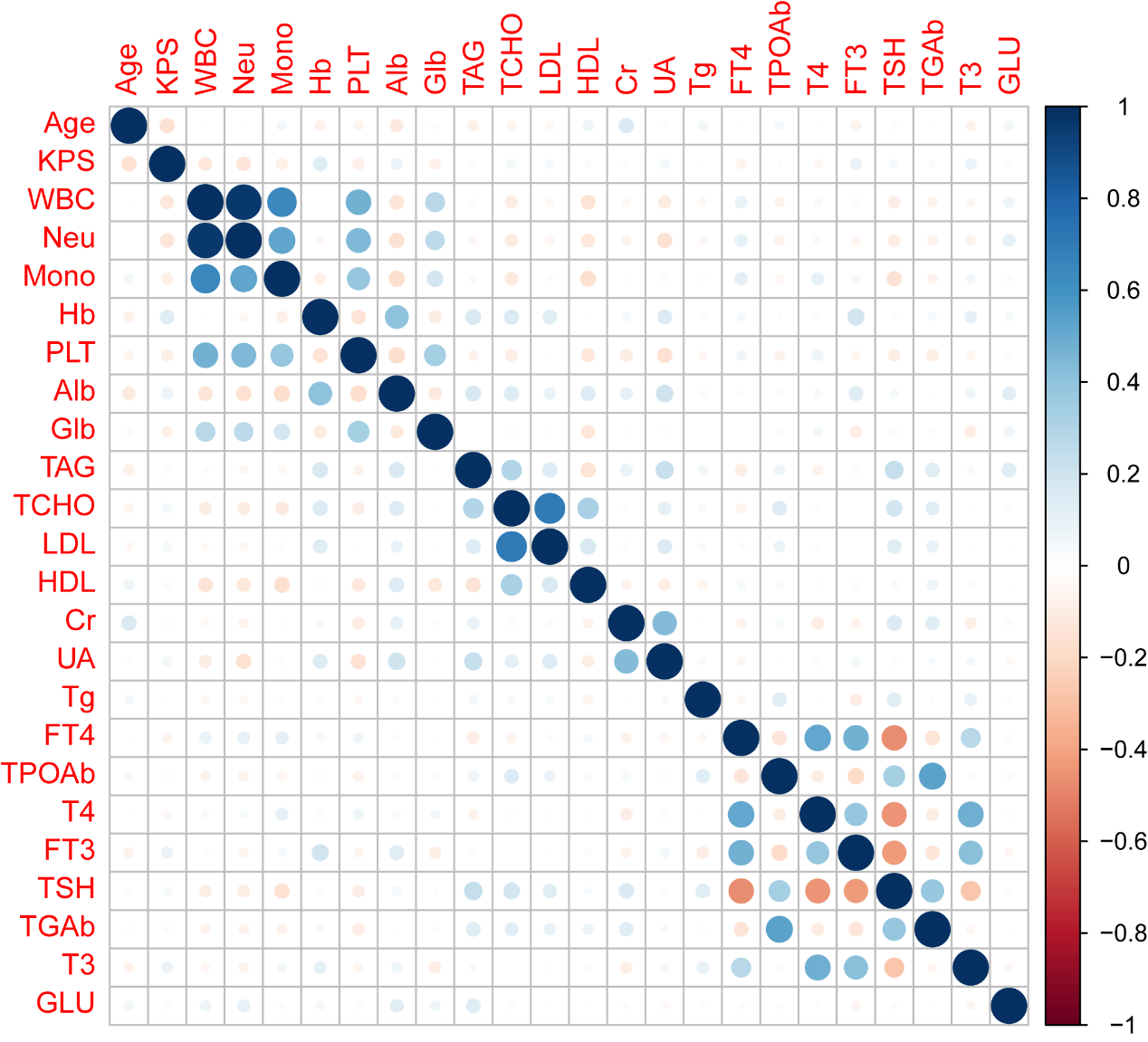


Supplementary Figure 1. Correlation heatmap between feature variables.
